# Reliable landmarks for precise topographical analyses of pathological structural changes of the ovine tibial plateau in 2-D and 3-D subspaces

**DOI:** 10.1038/s41598-017-18426-3

**Published:** 2018-01-08

**Authors:** Tamás Oláh, Jan Reinhard, Liang Gao, Lars K. H. Goebel, Henning Madry

**Affiliations:** 1 Center of Experimental Orthopaedics, Saarland University, Homburg, Germany; 2 Department of Orthopaedic Surgery, Saarland University Medical Center, Homburg, Germany

## Abstract

Selecting identical topographical locations to analyse pathological structural changes of the osteochondral unit in translational models remains difficult. The specific aim of the study was to provide objectively defined reference points on the ovine tibial plateau based on 2-D sections of micro-CT images useful for reproducible sample harvesting and as standardized landmarks for landmark-based 3-D image registration. We propose 5 reference points, 11 reference lines and 12 subregions that are detectable macroscopically and on 2-D micro-CT sections. Their value was confirmed applying landmark-based rigid and affine 3-D registration methods. Intra- and interobserver comparison showed high reliabilities, and constant positions (standard errors < 1%). Spatial patterns of the thicknesses of the articular cartilage and subchondral bone plate were revealed by measurements in 96 individual points of the tibial plateau. As a case study, pathological phenomena 6 months following OA induction *in vivo* such as osteophytes and areas of OA development were mapped to the individual subregions. These new reference points and subregions are directly identifiable on tibial plateau specimens or macroscopic images, enabling a precise topographical location of pathological structural changes of the osteochondral unit in both 2-D and 3-D subspaces in a region-appropriate fashion relevant for translational investigations.

## Introduction

Mapping of pathological structural changes such as occurring in osteoarthritis (OA) in translational models to a precise topographical location is critical for establishing connections between the underlying factors of this debilitating musculoskeletal disease that likely act in an integrated fashion^1,2^. Large animal models such as sheep provide for a unique experimental platform to identify mechanisms of OA development^3^, as their (stifle) joints reflect many key features of the human knee, including the presence of bicondylar distal femora, menisci, the axial alignment of the lower limb, and the relative sizes of the articulating partners^3–8^. The tibial plateau is frequently in the centre of OA, including the development of ill-defined OA cartilage lesions and osteophytes.

In the ovine model, such pathological osteochondral alterations may be examined at very high resolutions with micro-computed tomography (micro-CT), besides macro- and microscopic analyses. Micro-CT is capable of virtually separating the acquired samples into 2-dimensional (2-D) sections and allows for an objective measurement of the 3-dimensional (3-D) microarchitecture on a microscopic scale^9–13^, a possibility not feasible in clinical settings^10,11,13–17^. Moreover, 3-D image registration is available to aid with the problem of identical sample orientation^18,19^. So far, regions on the ovine tibial plateau were defined either visually (based on the presence of menisci^20^, separation into halves^21^ or thirds^22–24^), by mathematical modelling or using measured^25^ or projected grids^26^. However, objectively definable and reproducible regions based on stable, easily identifiable reference points are largely lacking. This further complicates inter-study comparisons to elucidate the structural basis for communications within the osteochondral unit.

The aim of the present study was to propose stable reference points reflecting anatomic landmarks of the ovine tibial plateau and to define regions, allowing for a convenient and accurate sample harvesting from identical locations. We hypothesized that such objectively recognizable reference points -visible both by macroscopic analysis and on micro-CT sections- can be defined on the ovine tibial plateau. We further hypothesized that these reference points can be used to divide the tibial plateaus into subregions relevant for translational investigations of OA with a high reproducibility. Next, using these subregions we described topographical patterns of the thickness distribution of the osteochondral unit and of pathological phenomena such as OA cartilage lesions and osteophytes in a fashion relevant for translational investigations. Our data show that reliable landmarks for precise topographical analyses of the ovine tibial plateau in 2-D and 3-D subspaces can be defined, allowing for a quick and convenient identification of pathological structural changes.

## Results

### Anatomical considerations and definition of landmark reference points and lines of the ovine tibial plateau

**Figure 1:**
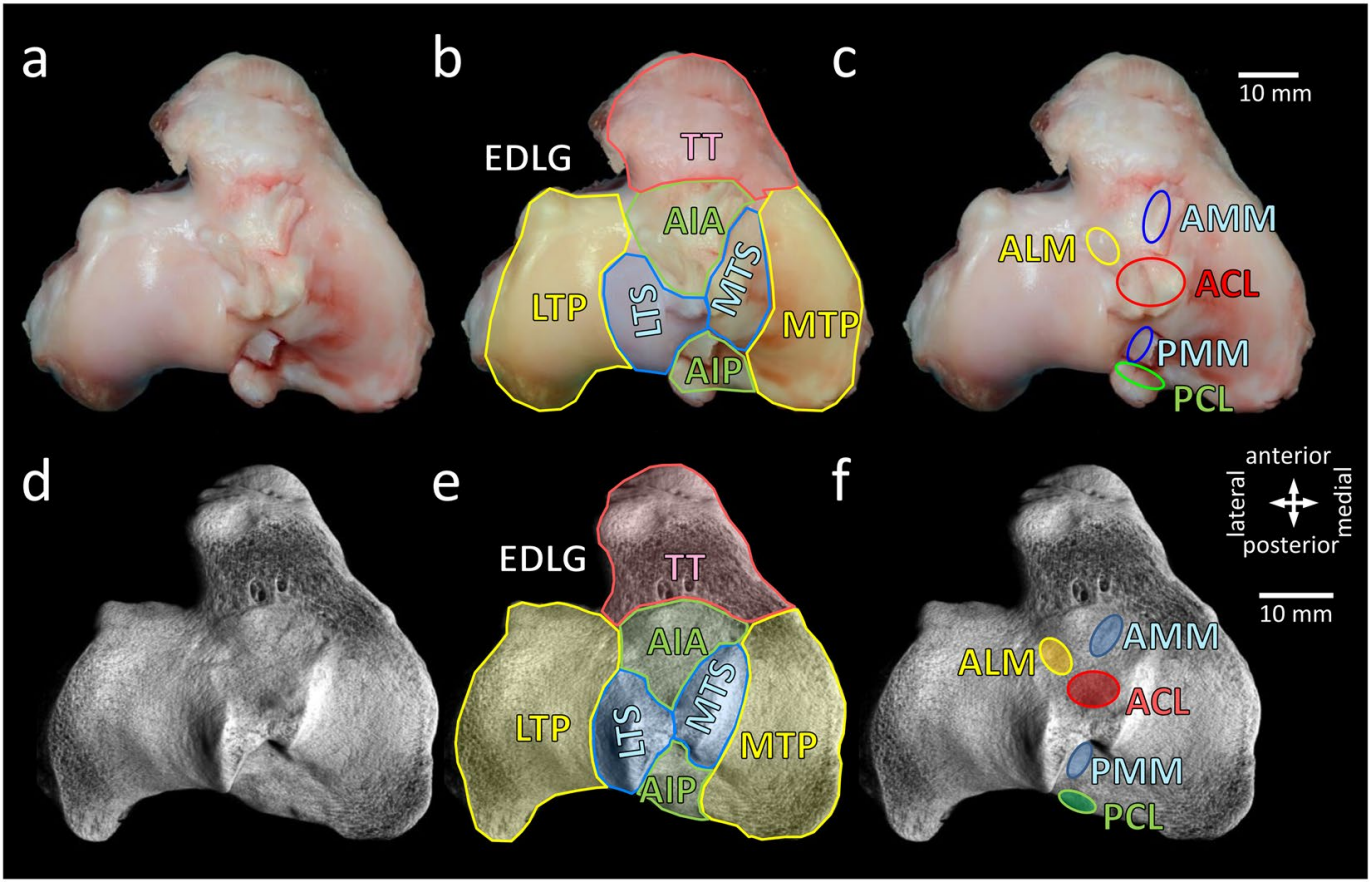
Anatomy of the ovine tibial plateau. **(a**–**c)** Macroscopic view and **(d**–**f)** 3-D reconstructed micro-CT images of a left ovine tibial plateau. **(b**,**e)** Qualitative description of the main individual areas of the entire tibial plateau. Note the prominent tibial tuberosity (TT) and groove of the extensor digitorum longus tendon (EDLG) on the anterior end. The shape of the medial (MTP) and lateral tibial plateau (LTP) is steeper and less oval compared to the human ones. Anterior to the medial (MTS) and lateral tibial spine (LTS) the area intercondylaris anterior (AIA) has a notable prominence. **(c**,**f)** Insertion sites of the crucial ligaments (ACL, anterior cruciate ligament; PCL, posterior cruciate ligament) and the menisci (ALM, anterior horn of the lateral meniscus; AMM, anterior horn of the medial meniscus; PMM, posterior horn of the medial meniscus) on the tibial plateau. Scale bars are identical for panels a–c and d–f respectively. Directions are identical for all panels. Abbreviation not mentioned elsewhere: AIP, area intercondylaris posterior.

The medial and lateral tibial plateaus of the ovine proximal tibia are of an irregular oval shape with a substantial posterior slope (Fig. 1). A prominent groove of the extensor digitorum longus tendon lies anterior to the lateral tibial plateau. Of note, the lateral tibial spine arises more posterior than the medial tibial spine, both fusing in the area between them in a saddle-like prominence. The area intercondylaris anterior, located in front of these prominent tibial spines, has a notable elevation. More anterior, a distinct tibial tuberosity is present. The posterior-anterior length of the medial tibial plateaus, determined on series of coronal micro-CT 2-D sections between the posterior edge of the medial tibial plateau and the anterior edge of the lateral tibial plateau close to the groove of the extensor digitorum longus tendon, had an average length of 32.7 ± 0.3 mm (n = 18).

**Figure 2:**
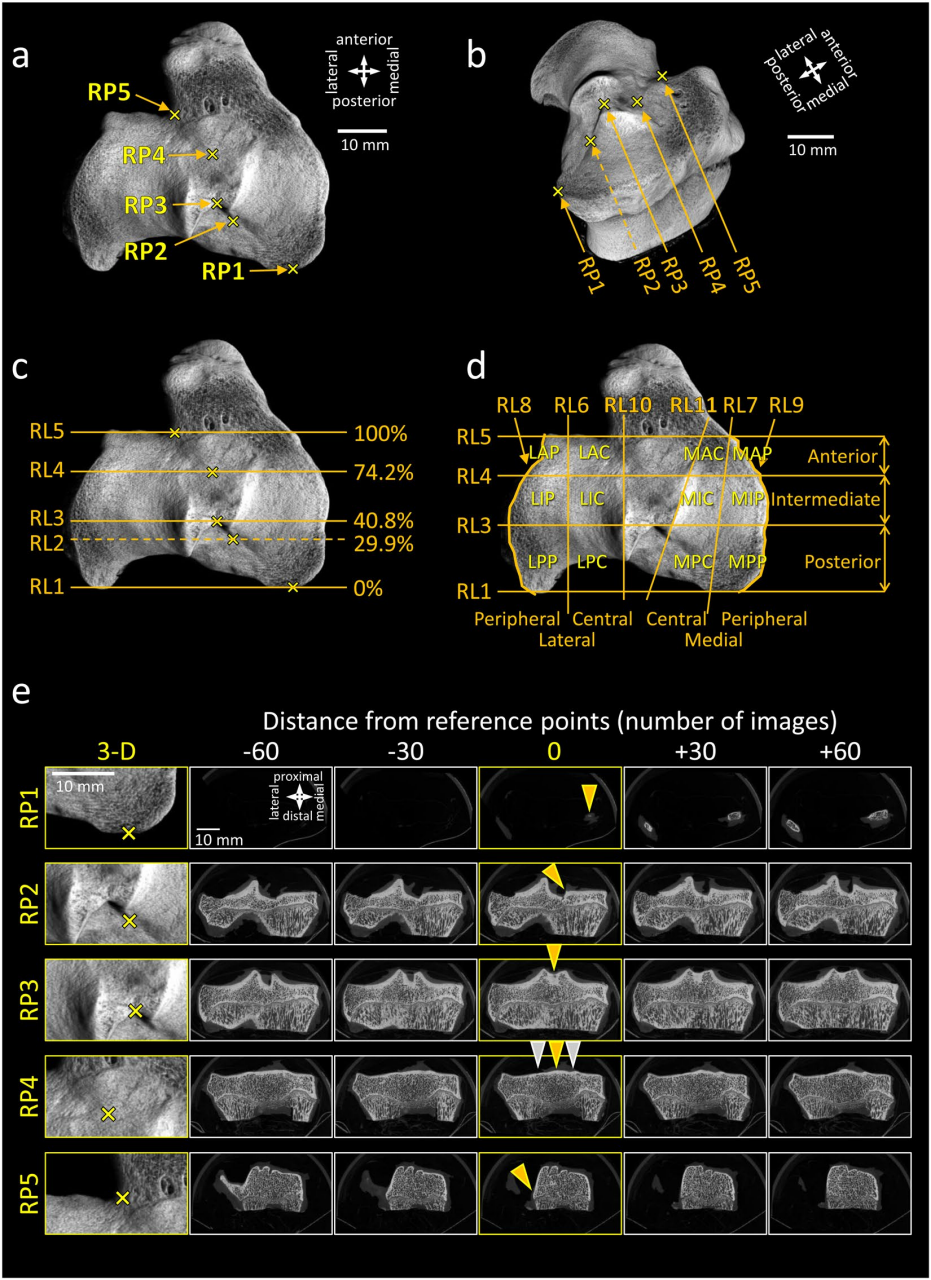
Newly defined reference points, reference lines and subregions on the left ovine tibial plateau. **(a)** Location of the five reference points (RP1-5). **(b)** 3-D image of the same sample from a different point of view, showing the reference points. **(c)** Reference lines (RL1-5) defined by the reference points. Percentages are the relative positions of the RLs compared to the length of the tibial plateau (mean values of n = 18 specimens). **(d)** 6-6 subregions defined by the reference points on the lateral and medial tibial plateau. Note: RP2 was not used for defining the regions. The reference points and reference lines, forming the boundaries of the regions are described in Table 1. (**e**) Higher magnification 3-D images (directions are identical to that of panel a) and consecutive micro-CT image slices of a left tibial plateau of a sheep serving as a key for determining the reference points. 5-5 images showing the bony structures at and around the 5 reference points are displayed. 0 position (middle column) is the image number where the reference point can be found (indicated by yellow arrowheads). Other images are also shown posterior (−60 and −30 images distance from the reference point; equals to −0.53 and −1.06 mm, respectively; first two columns) and anterior (+30 and +60 images; last two columns) from the reference images for easier identification of the RPs. Yellow arrowheads are pointing to the reference points. Grey arrowheads are pointing to the tibial spines to show the position where they become smaller than the bony prominence of the area intercondylaris anterior. Scale bars and directions are identical for panels a, c, and d.

Based on these anatomical observations, 5 bony reference points (RP1-5) were defined to serve as landmarks (Fig. 2, Table 1). Reference point 1 (RP1) reflects the posterior end of the medial tibial plateau. RP2 is the posterior beginning of the medial tibial spine. RP3 refers to the point (saddle) where the lateral and medial tibial spines fuse. RP4 is the posterior beginning of the elevation of the area intercondylaris anterior. RP5 mirrors the anterior end of the lateral tibial plateau, similar to the groove of the extensor digitorum longus tendon. The relative position of the five individual reference points were at 0%, 29.9 ± 0.7%, 40.8 ± 0.8%, 74.2 ± 0.8% and 100% of the total posterior-anterior length (n = 18). Their relative position remained constant between the individual samples, as judged by standard errors of less than 1% (n = 18; Fig. 2c).

**Table 1:** Reference points (RP) and lines (RL) used for dividing the ovine tibial plateau into subregions based on 2-D micro-CT images.

Reference point/line	Description	Comment
RP1	Posterior edge of the medial tibial plateau.	First image of the data set showing the bony tibial plateau. Definition: 0% of the tibial plateau length.
RP2	Posterior beginning of the medial tibial spine as position where the medial tibial spine starts to emerge as seen from posterior.	Although the lateral tibial spine starts more posterior, this beginning of the medial tibial spine can be better identified.
RP3	Position of the point (saddle) where the medial tibial spine fuses with the lateral tibial spine as seen from posterior.	The point where both tibial spines fuse is easily recognizable because of its flat U-form, comparable to a saddle.
RP4	Position where the bony prominence of the area intercondylaris anterior starts to emerge above the height of the anterior end of both tibial spines.	This point is located similar to the anterior beginning of the medial tibial spine.
RP5	Anterior edge of the lateral tibial plateau. Anterior beginning of the bony part of the lateral tibial plateau, similar to the bony groove of the extensor digitorum longus tendon.	Definition: 100% of the tibial plateau length. This is not the last image of the data set, as the prominent tibial tuberosity (where the patellar tendon attaches) follows more anteriorly, outside of both tibial plateaus.
RL1	Line parallel to the coronal section of the tibial plateau, crossing RP1.	
RL2	Line parallel to the coronal section of the tibial plateau, crossing RP2.	RL2 is not needed for determining the regions.
RL3	Line parallel to the coronal section of the tibial plateau, crossing RP3.	
RL4	Line parallel to the coronal section of the tibial plateau, crossing RP4.	
RL5	Line parallel to the coronal section of the tibial plateau, crossing RP5.	
RL6	Line connecting the halving point of RL3 between the lateral tibial spine and external edge of the lateral tibial plateau, with the halving point of RL4 between the lateral tibial spine and the external edge of the lateral tibial plateau.	RL6 or RL7 lines can be made visible in the micro-CT analysing programs (e.g. CTan from Bruker Micro-CT) as defining small regions of interests (ROIs) on the fixed points of the RLs described here, and performing inter- and extrapolation through the whole image set.
RL7	Line connecting the halving point of RL3 between the medial tibial spine and the external edge of the medial tibial plateau, with the point where RL5 meets with the edge of the medial tibial plateau.	
RL8	Peripheral margin, lateral tibial plateau.	
RL9	Peripheral margin, medial tibial plateau.	
RL10	Line spanning along the lateral tibial spine, representing the central margin of the lateral tibial plateau.	RL10 or RL11 lines can be made visible in the micro-CT analysing program as defining small ROIs on the lateral or medial tibial spines and performing inter- and extrapolation through the whole image set.
RL11	Line spanning along the medial tibial spine, representing the central margin of the medial tibial plateau.	

Subsequent, 5 lines were projected through each individual reference point, parallel to each other (Table 1; Fig. 2c). These reference lines (RL1-5) divide the both medial and lateral tibial plateaus into 3-3 regions. In detail, the posterior region lies between RP1 and RP3, the intermediate region between RP3 and RP4, and the anterior region between RP4 and RP5. To allocate pathological changes (e.g. OA lesions, osteophytes) even more accurately, 6 additional reference lines (RL6-11) which split the above defined regions into their halves were introduced. These lines divide both tibial plateaus into 6 individual subregions.

**Figure 3:**
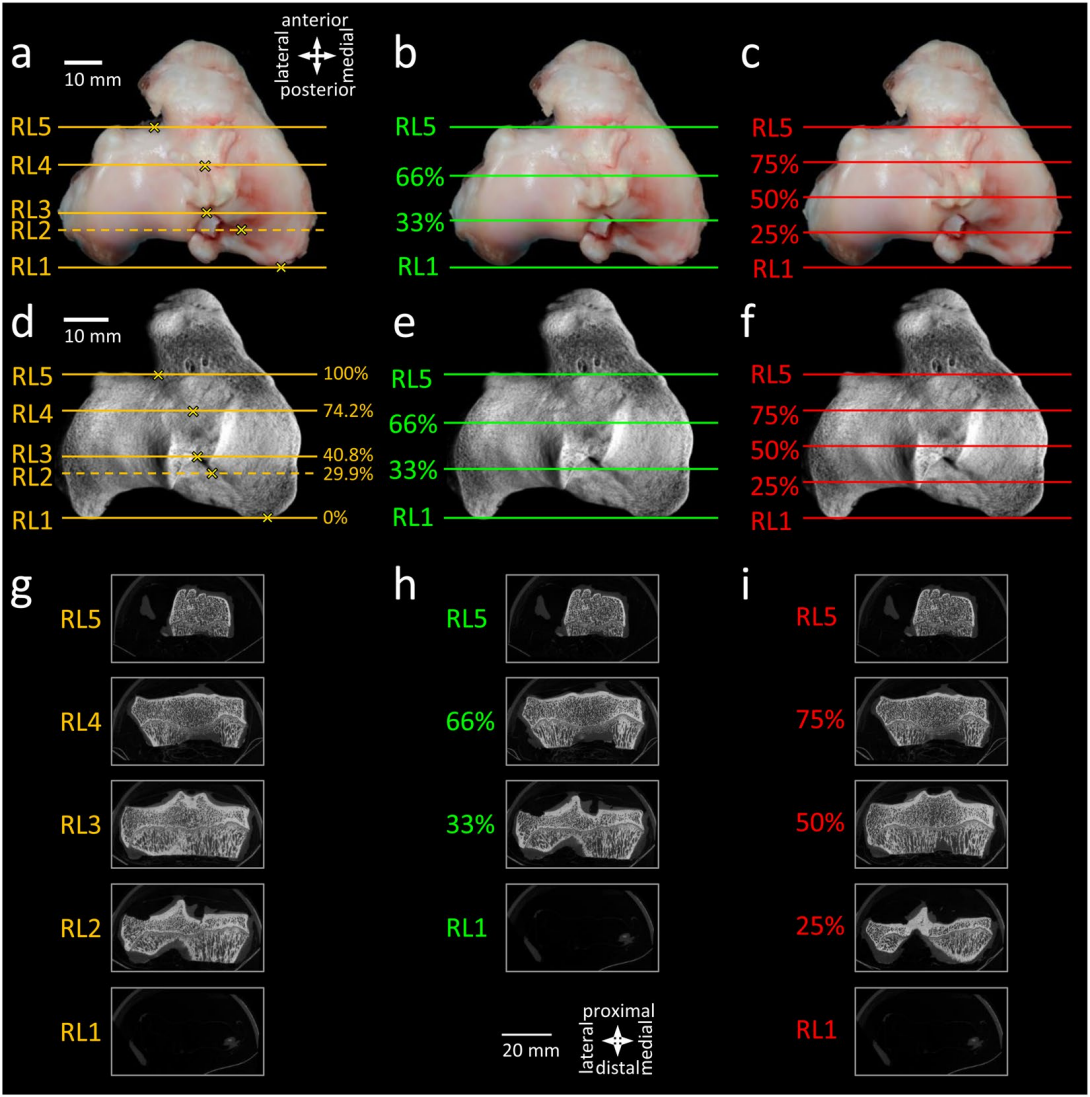
Comparison of subregion systems of the ovine tibial plateau. **(a**–**c)** Macroscopic images and **(d**–**f)** 3-D reconstructed micro-CT images of a left tibial plateau, showing the reference lines defining the boundaries of the regions. **(g**–**i)** 2-D micro-CT slices corresponding to the reference lines. Subregion systems using **(a**,**d**,**g)** the newly defined reference lines described in the present manuscript, and a division of the tibial plateau into **(b**,**e**,**h)** thirds and **(c**,**f**,**i)** quarters by using RL1 and RL5 as the boundaries of the tibial plateau. Directions are identical for panels a–f, and g–i, respectively. Scale bars are identical for panels a–c, d–f, and g–i, respectively.

If equal-sized subregions have to be defined, the reference points RP1 and RP5 may help to accurately placing a grid onto the tibial plateau thus separating the tibial plateau length into defined percentages (Fig. 3). Image numbers corresponding to (clinically relevant) thirds (33%, 66%) or quarters (25%, 50%, 75%) of the tibial plateau can conveniently be identified in a series of 2-D micro-CT slices by using the following formula (1):

1 $${{\bf{N}}}_{{\bf{p}}}={{\bf{N}}}_{{\bf{RP1}}}+[({{\bf{N}}}_{{\bf{RP5}}}-{{\bf{N}}}_{{\bf{RP1}}})\,{\boldsymbol{\ast }}\,{\bf{p}}/{\bf{100}}]$$

where N_p_ is the image number corresponding to the micro-CT slice of the desired percentage, N_RP1_ is the image number corresponding to the micro-CT slice of RP1, N_RP5_ is the image number corresponding to the micro-CT slice of RP5, and p is the percentage.

### Sensitivity of the relative distance of the reference points to rotation of the reconstructed image and 3-D image registration

**Figure 4:**
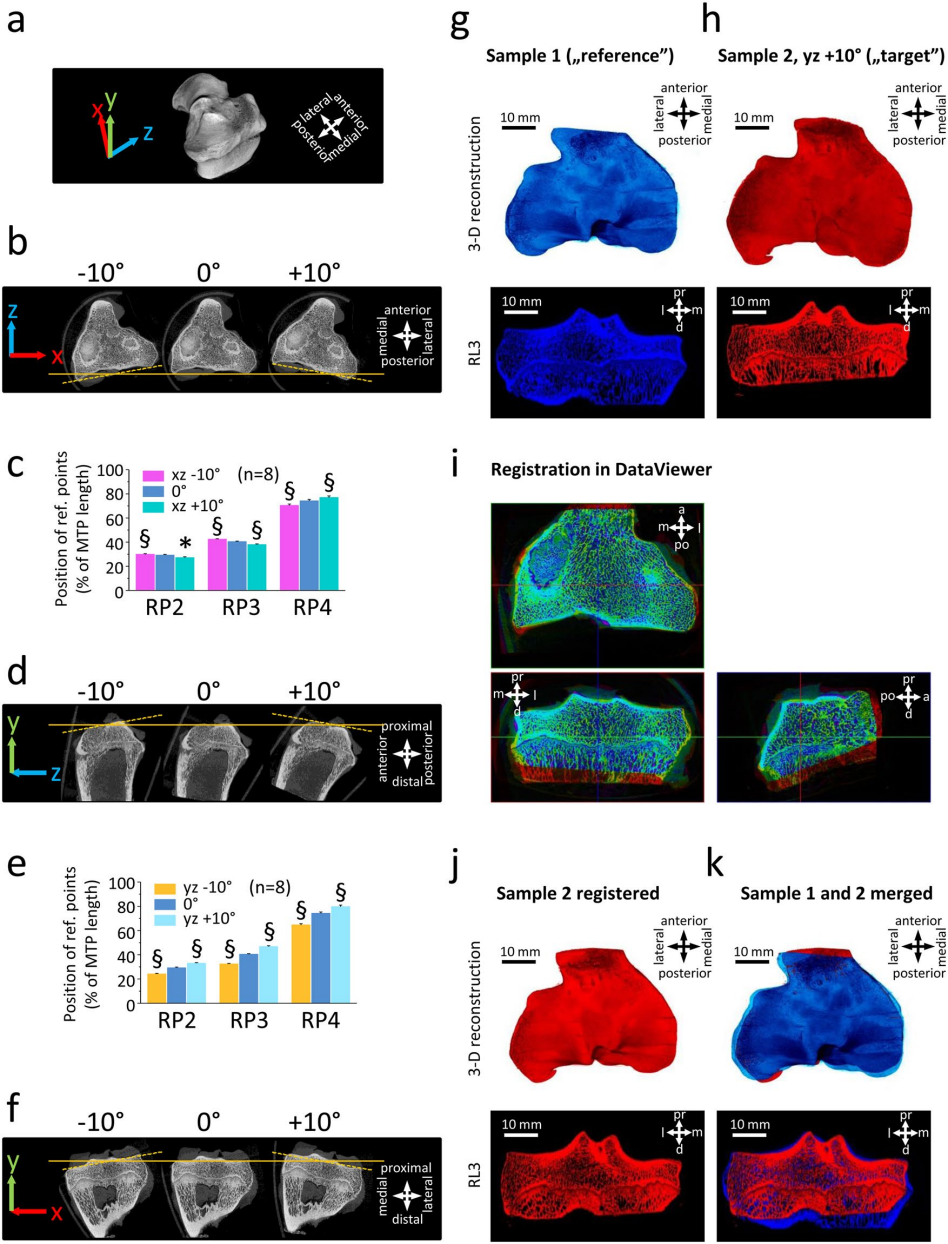
Sensitivity of the relative distance of the reference points to the rotation of the samples. **(a)** 3-D reconstructed image of a sheep left tibial plateau (already presented on Fig. 2b) showing the XYZ directions used for determining the planes for the rotations. Direction and colour of the arrows on panels b, d, and f are in congruence with that of panel a. **(b)** Rotation of a representative sample to −10°and +10° clockwise in the axial (xz) plane. 0° shows the original position of the sample. **(c)** Position of the reference points (% of the medial tibial plateau (MTP) length) in n = 8 samples rotated to −10° and +10° clockwise in the axial plane. **(d)** Rotation of a representative sample to −10° and +10° clockwise in the sagittal (yz) plane. 0° shows the original position of the sample. **(e)** Position of the reference points (% of the medial tibial plateau length) in n = 8 samples rotated to −10° and +10° clockwise in the sagittal plane. **(f)** Rotation by the coronal (xy) plane was not tested because, being the original slicing plane, it does not affect the relative position of the RPs. *P < 0.05, ^§^ *P* < 0.01; rotated vs. 0°. **(g**–**k)** 3-D image registration in DataViewer provides a complementary solution to achieve identical rotation of the samples. **(g)** Sample 1 (blue) with appropriate rotation was used as a reference image. On the bottom subpanel a micro-CT slice taken from the position of RL3 is shown. **(h)** Sample 2 (red) was rotated by +10° in the sagittal (yz) plane to achieve an inappropriate rotation as an example. **(i)** Image sets were registered in DataViewer as shown on images from the program. **(j)** After registration the alignment of Sample 2 was comparable to that of Sample 1. **(k)** Merged image of Sample 1 and 2 after registration. Scale bars and directions are identical for panels g, h, j, k. Abbreviations: a: anterior, d: distal, l: lateral, m: medial, pr: proximal, po: posterior.

When samples were rotated (Fig. 4) in the axial plane (xz), the relative distances of the reference points slightly changed (ranging from 0.7 ± 0.2% to 3.8 ± 0.5%). One way repeated measures analysis of variance (RM ANOVA) revealed these changes as statistically significant (*P* < 0.05 or *P* < 0.01) compared to the original rotation. When samples were rotated in the sagittal plane (yz), the alterations in the relative distances of the reference points were more prominent and statistically significant (ranging from 3.8 ± 0.4% to 9.5 ± 0.4%; *P* < 0.01 vs. original rotation in all cases). Rotation in the coronal plane (xy) was not tested, because this rotation – being the original plane of the virtual slices of the samples - does not alter the image numbers and the relative distance of the reference points.

Next, 3-D image registration in the DataViewer program was applied as an aid to achieve identical rotation of the samples when the manually set rotational parameters do not give satisfying results (Fig. 4g–k). In the example shown here, a correctly rotated image set of a sample was chosen as a reference, and another sample was rotated by +10° in the sagittal (yz) plane to serve as a target volume. After a true 3-D rigid registration in DataViewer, the alignment of the two samples was identical as assessed by visual inspection of the new 2-D image sets and the 3-D reconstructed volumes.

### Intraobserver reliability of the reference points

**Figure 5:**
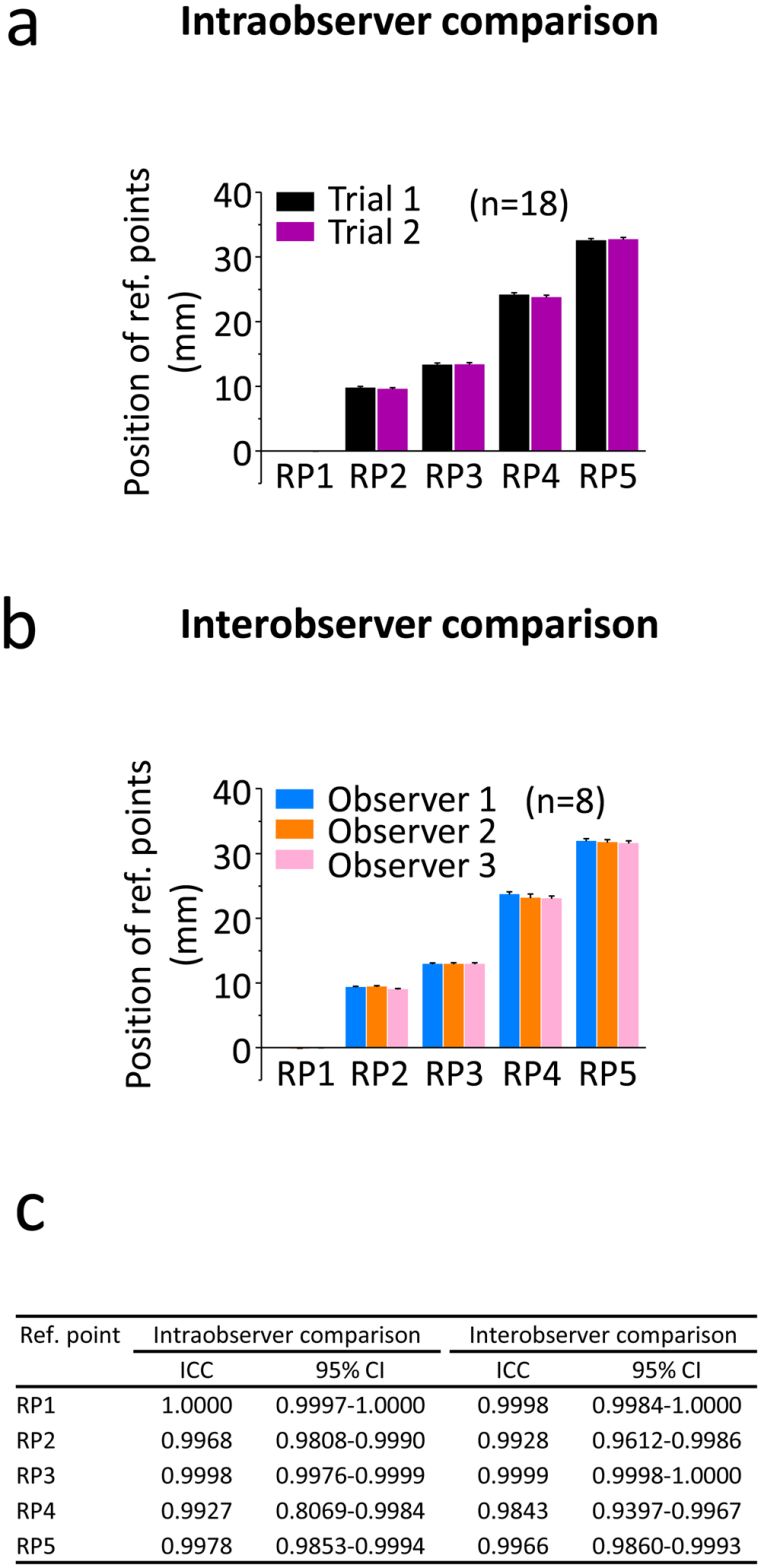
Intra- and interobserver comparison for testing the reliability of the reference points. **(a)** Intraobserver variability of the reference points. The same observer determined the localization of the 5 reference points on the same image sets of 18 tibial plateau samples twice with 3 weeks difference between Trial 1 and 2. **(b)** Interobserver variability of the reference points. The same 8 image sets of left tibial plateaus were evaluated by 3 individual observers to find the 5 reference points. **(c)** Intraclass correlation coefficients (ICC) and 95% confidence intervals (CI) of the comparisons.

Between Trial 1 and Trial 2 the difference was ranging from 0.02 ± 0.01 mm at RP1, to 0.39 ± 0.07 mm at RP4 (n = 18 samples) (Fig. 5). When these errors were calculated in percentages of the total length of the tibial plateau, the greatest error (at RP4) was 1.2 ± 0.2%, well within the 5% acceptance range of the preliminary criterion. The calculated intraclass correlation coefficients were always higher than 0.99.

### Interobserver reliability of the reference points

Comparing the three observers, the lowest error of the landmarks was 0.002 ± 0.01 mm at RP3 between Observer 1 and 2, and the highest was 0.65 ± 0.06 mm at RP4 between Observer 1 and 3 (n = 8 samples) (Fig. 5). Compared to the total length of the tibial plateaus, the maximal error was lower than 5% in all cases, 2.05 ± 0.19% being the highest at RP4 between Observer 1 and 3. Intraclass correlation coefficients were always higher than 0.98.

### Assessment of the usability of the topographic regions defined by the reference points for a sample harvesting

**Figure 6:**
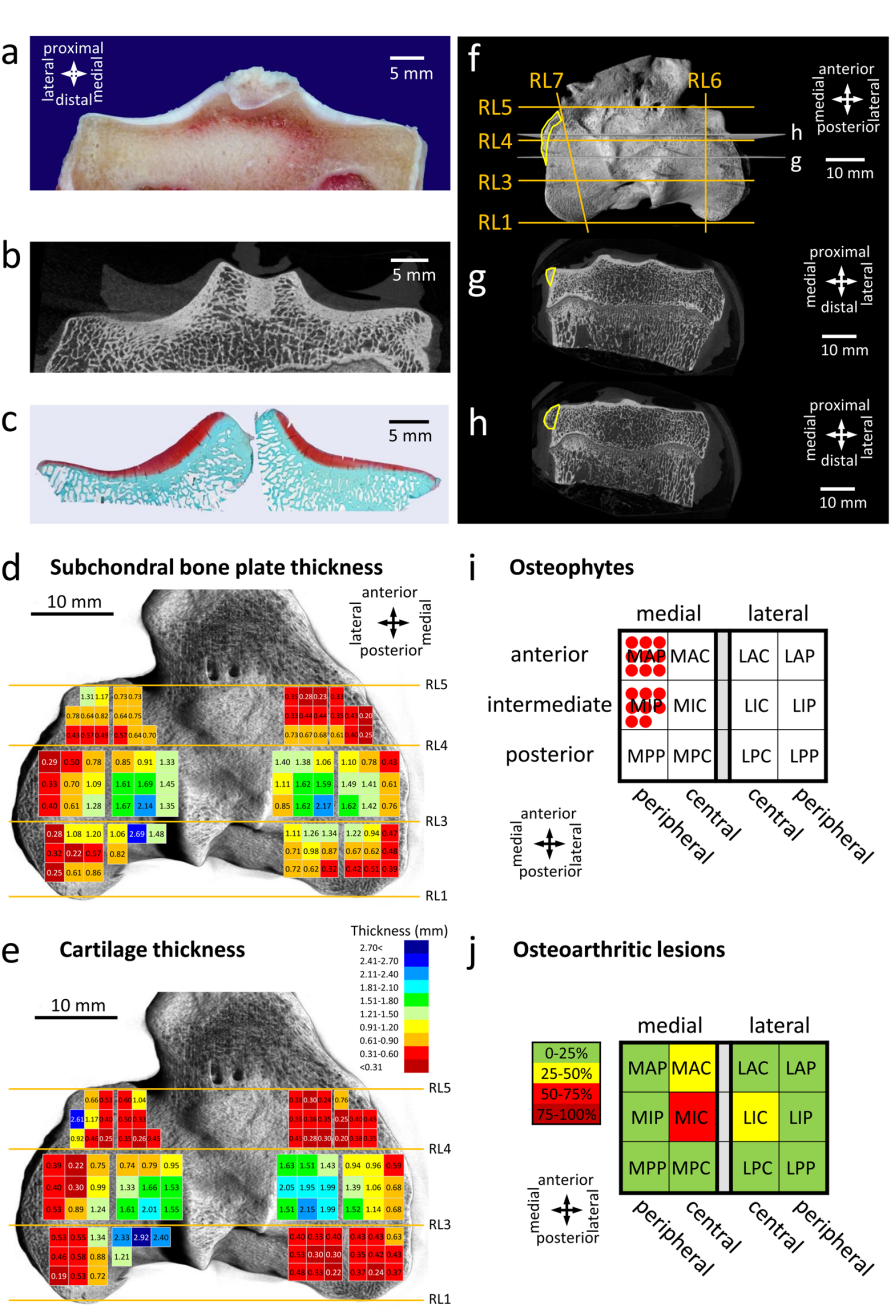
Evaluation of the usability of the newly defined regions. **(a)** Representative macroscopic image, **(b)** a 2-D micro-CT section, and **(c)** a Safranin O/fast green-stained histological section from the intermediate region of a left tibial plateau, showing the usability of the newly defined regions for sample harvesting for histological staining. Directions are identical for panels a-c. Map of the spatial distribution of the thickness of **(d)** the subchondral bone plate and **(e)** the articular cartilage across the topographic regions of a representative normal ovine tibial plateau marked by the reference lines. Directions and colour code are identical for both panels. **(f)** Representative 3-D reconstructed micro-CT image of a right tibial plateau showing the subregions (note that for clarity, only the relevant reference lines are shown). A large osteophyte, extending to multiple regions is marked with yellow. The images corresponding to the grey slicing planes labelled by g and h are shown at panels g and h. **(g)** Example of a 2-D image with osteophyte from the intermediate region. **(h)** Example of a 2-D image with osteophyte from the anterior region. **(i)** Distribution of osteophytes on the right tibial plateau. A red circle shows the presence of an osteophyte in the evaluated subregion in one sample (n = 9). (**j)** Distribution of OA lesions on the right tibial plateau (schematic representation). Evaluation was based on the average of n = 9 India ink stained samples. Colour code shows the percentage of the OA lesion-covered areas within the subregions. Abbreviations of the names of the subregions on panels i and j are identical to that of Fig. 2d.

Since one of the primary goals was to provide a solution for the difficult problem of obtaining a reproducible sample harvesting from identical regions of multiple tibial plateau specimens, the usability of the topographic regions defined by macroscopy and the reference points on micro-CT were then assessed (Fig. 6a–c). As a case study, the intermediate region of both tibial plateaus following macroscopic observation was investigated. Its boundaries, RP3 and RP4, were macroscopically identified on the samples, and histological sections were taken from between them, and stained with Safranin-O/fast green. The exact location of the histological sections within the region was more precisely identified with the aid of micro-CT. Micro-CT 2-D images within this region were retrieved, and images that directly corresponded to the histological sections were identified, thus enabling to locate a histological section on a (macroscopic) area of interest.

### Assessment of usability of the regions defined by the reference points for a topographic examination of the thickness of the articular cartilage and subchondral bone plate

Next, the thicknesses of the articular cartilage and the subchondral bone plate in individual measurement points (n = 98) in the newly defined topographic regions of the tibial plateau (medial: n = 52, lateral: n = 46) were examined by micro-CT in a randomly selected normal control sample (Fig. 6d,e). The individual thickness values of the articular cartilage ranged from 0.20 to 2.15 mm in the medial and from 0.19 to 2.92 mm in the lateral tibial plateau. The individual thickness values of the subchondral bone plate were from 0.20 to 2.17 mm in the medial and from 0.22 to 2.69 mm in the lateral tibial plateau. Their specific topographical pattern was similar: both the articular cartilage and the subchondral bone plate were thinner (<1 mm) in the (submeniscal) peripheries, while both were thicker in the central regions (>1 mm) of both tibial plateaus.

### Mapping of pathological osteophyte development to the newly defined topographic regions

Following induction of OA by transection of the anterior root of the medial meniscus, the samples were examined by micro-CT for the presence of osteophytes after 6 months *in vivo* in the newly defined topographic regions (Fig. 6f–i). Osteophytes (n = 9) were identified in each of the 9 samples. They were always located in the medial intermediate peripheral and medial anterior peripheral regions. Most osteophytes (n = 8) extended over both subregions. No osteophytes were identified in other regions.

### Assessment of usability of the topographic regions defined by the reference points for examination of macroscopic OA changes

The macroscopic topographic regions on the sheep tibial plateau defined by the reference points were examined for osteoarthritic changes following OA induction (n = 9). India Ink staining revealed areas affected by OA, and the extensions of each area within the individual subregions was evaluated. The largest areas subjected to OA were located on the medial intermediate central (50–75% of its area), the medial anterior central, and the lateral intermediate central regions (25–50% of their areas). Other subregions were only slightly affected by OA changes (0–25% of their areas) (Fig. 6j).

### Value of the reference points for landmark-based 3-D image registration

**Figure 7:**
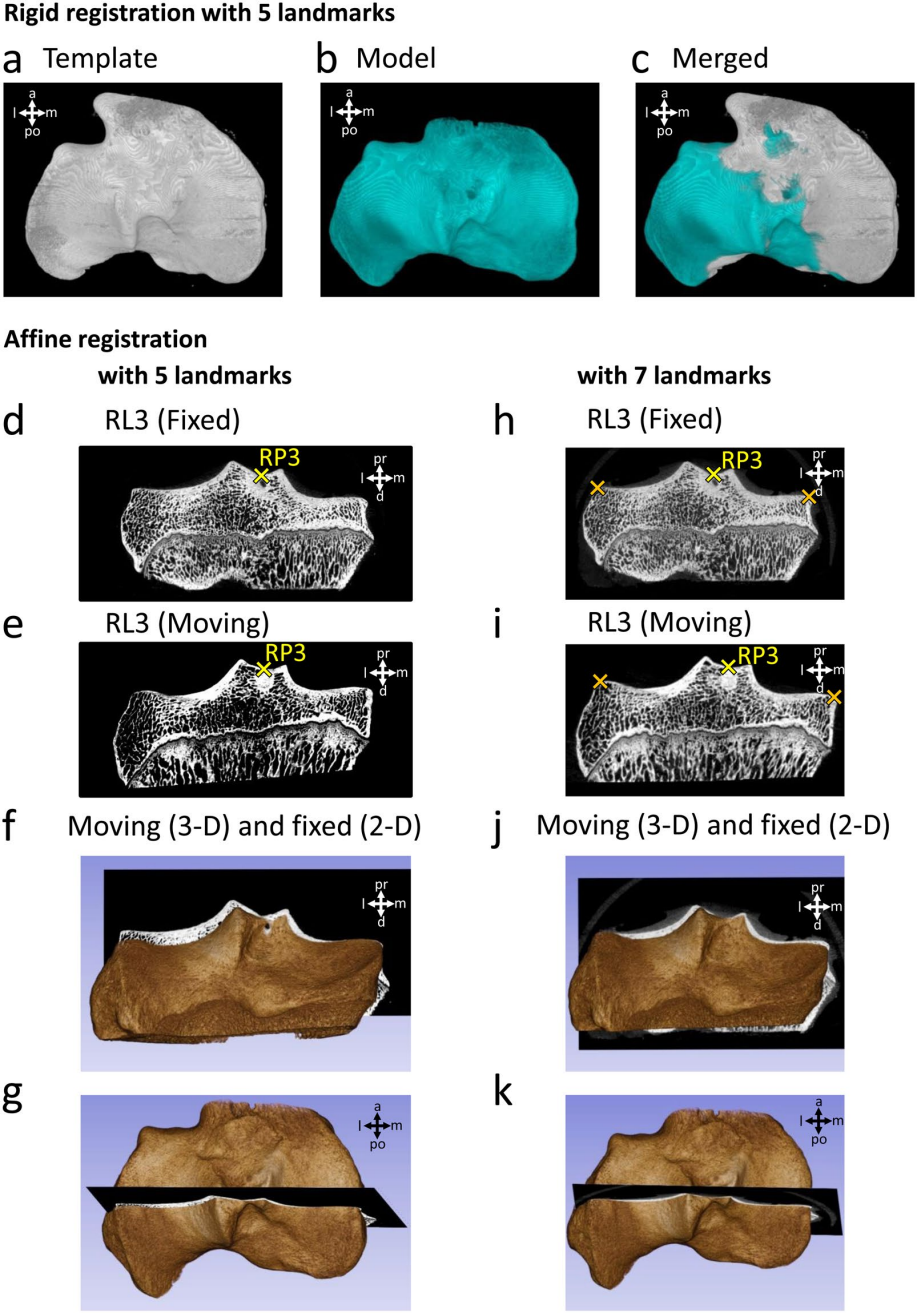
Assessment of the usability of the reference points for landmark-based 3-D registration. **(a**–**c)** Landmark-based rigid registration of two tibial plateau samples in the Fiji software, using RP1-5 as landmarks. 3-D view of the **(a)** template and **(b)** model image after registration. **(c)** Merged view of the template and model images. Landmark-based affine registration of the same two samples in the 3D Slicer software, **(d**–**g)** using only RP1-5 as landmarks, and **(h**–**k)** using additional 2 landmarks (labelled with orange crosses) to RP1-5, placed on the medial end of the medial tibial plateau and the lateral end of the lateral tibial plateau on the 2-D section corresponding to RL3. Images show the transformed samples after registration. Example micro-CT images corresponding to RL3 of the samples set as the **(d**,**h)** fixed and **(e**,**i)** the moving volume. **(f**,**j)** Posterior and **(g**,**k)** top view of the two samples merged after registration. The 3-D volume shows the sample set as the moving volume, and the 2-D micro-CT section shows RL3 of the sample set as the fixed volume. Abbreviations: a: anterior, d: distal, l: lateral, m: medial, pr: proximal, po: posterior.

Finally, the usability of the reference points for landmark-based 3-D image registration was assessed. With the aim of testing both rigid and affine transformation algorithms, the Fiji and the 3D Slicer programs were selected (Fig. 7). RP1-5 were defined manually on 2-D micro-CT image sets of 2 randomly chosen normal knees in both programs, and their 3-D registration was performed. After visual inspection of the resulting 2-D and/or 3-D image sets, the overlap of the two samples was determined to be satisfactory with both transformation methods. Two additional landmarks, placed on the medial end of the medial tibial plateau and the lateral end of the lateral tibial plateau on the 2-D section corresponding to RL3 of the samples (Fig. 7h,i) further increased the accuracy of the affine registration with 3D Slicer.

## Discussion

An objective definition of bony reference points on the ovine tibial plateau based on 2-D sections of micro-CT images which may serve as standardized landmarks for landmark-based 3-D image registration that can be translated to the macroscopic aspect has not, to the best of our knowledge, been performed to date. In the present study, we provide distinct definitions of 5 new bony reference points of the ovine tibial plateau based on anatomical landmarks. These 5 reference points are easily identifiable macroscopically and on 2-D micro-CT sections. A characterization of their identification revealed high intra- and interobserver reliabilities. Based on these points, each tibial plateau can be divided into 6 subregions, allowing for a precise definition of the topographical location of sample harvesting. These subregions also allow topographical thickness measurements of components the osteochondral unit. Finally, topographical allocations of pathological phenomena such as OA cartilage lesions and osteophytes in a fashion relevant for translational investigations are possible using this approach.

A precise and reproducible definition of regions affected by pathologies based on stable landmarks may aid translational investigations, as the tibial plateau is often affected by OA or injuries to its articular cartilage^27^, attachments of cruciate ligaments^28^ and menisci^29^. Both intra- and extraobserver comparisons revealed an excellent reproducibility of locating the 5 reference points on coronal 2-D micro-CT sections, and the length of the medial tibial plateau is in good accordance with previously published values^7^. Judged from the errors and intraclass correlation coefficients of the comparisons, RP4 (protrusion of the area intercondylaris anterior) was relatively more difficult to identify compared to the other reference points. Yet, it remained within the pre-defined 5% error-acceptance range. The significant differences observed during the test for the rotation of the samples calls attention to the sensitivity of the relative distance of the reference points - and thus the relative proportions of the regions defined by them. Therefore, cautious and consistent rotation of the reconstructed micro-CT image sets is highly recommended prior to saving the coronal section view slices. A correct rotation is especially important in the sagittal plane, where a 10° change in rotation may translate into a near 10% change in the relative positions of RP3 and RP4. Importantly, this sensitivity to rotation serves as a valuable tool by using the relative distances of the reference points as indicators of the identical rotation of the images. When the manual rotation of a particular sample is complicated, 3-D image registration methods may give satisfactory results to achieve identically rotated image sets.

Definition of clinically relevant reference lines based on the anatomical landmarks is important for translational investigations. The tibial plateau may be divided into an anterior, intermediate and posterior region^30^. We aimed to provide precise definitions for these lines along 0, 33.3, 66.6 and 100% of the tibial plateau length, based on the proposed formula. They thus can be used to define the precise location of a pathology on corresponding macroscopic images. *Vice versa*, a macroscopic alteration can be precisely mapped to its corresponding subchondral bone micro-CT section, together with the appropriate histological image.

The presented subregion system permits a relatively constant and reproducible determination of the localization of cartilaginous OA lesions and osteophytes, together with the possibility of accurately plotting a map of the thicknesses of the articular cartilage and subchondral bone plate based on standardized measurement points within the subregions. The cartilage and the subchondral bone plate were thickest in the centre, and thinnest in the periphery of both the medial and lateral tibial plateaus, similar to previous data where cartilage thickness was measured with a needle penetration method at lower resolution^26^. OA lesions were mostly present in the medial anterior and intermediate central and the lateral intermediate central subregions, reflecting areas not covered by the menisci. Osteophytes always occurred in the periphery of the anterior and intermediate regions of the medial tibial plateaus, similar to other translational^23,31^ and clinical studies^32^. Previously, the ovine tibial plateau has been divided into regions based on distinguishing the central and peripheral areas^21^; meniscus covered and not covered regions^20^; inner, middle, and outer thirds^22^; cranial, middle and caudal thirds (each middle third was further divided into axial and abaxial aspects)^23,24^ as well as grids containing 9^25^ or 26 individual subregions^26^. Comparable regions were established on the human tibial plateau, for example by dividing it into meniscus covered and not-covered regions^33^, or in concentric zones (border and centre rings)^34^. Other studies divided the plateau into three^34^ or nine regions^35,36^, 4 quadrants^31^, 11 areas in the largest possible elliptical region of interest (ROI)^37^ or into 16 quadrants each (4 rows, 4 columns)^38^. Wirth and Eckstein provided a valuable MRI-based algorithm for the clinically increasingly important regional analysis of femorotibial cartilage thickness based on a central cylinder and 4 additional regions in radial arrangement^30^. Although all of these systems were reported to be usable for macroscopic observations, reproduction is complicated due to the requirement of specific equipment, complicated mathematical modelling or custom-made programs, or the subjectivity of the boundaries of the regions. Moreover, boundaries visible on 2-D micro-CT sections have not been described so far. The three proposed systems of subregions – either based directly on the reference points or on the calculations of the thirds or quarters of the tibial plateau length based on the stable landmarks RP1 and RP5 – have a significant advantage: they can be directly recognized or accurately determined both during micro-CT analysis and macroscopic investigations including sample harvesting. From an anatomical point of view, definition of these landmarks aids in identifying such anatomical landmarks on macroscopic images, which may be distorted by remaining soft tissue attachments following preparation (e.g. cruciate ligaments, meniscal roots).

The proposed system of subregions allows maximizing the comparability of physical specimens such as histological sections, thus reducing the inter-individual variability deriving from possible incongruent sample harvesting (different locations of different tibial plateau specimens). Although registering 2-D images to 3-D volumes^39–41^ allows for tracing back the original location of a 2-D histological section in a 3-D micro-CT dataset, it might be subjected to a considerable failure rate^42^, and alone may not ensure the comparability of the sections between different specimens, for which a congruent sample harvesting from standardized regions provides a robust and safe solution.

The described reference points and workflow is in principle compatible with the human tibial plateau, considering the anatomical differences: In humans, both medial and lateral tibial plateaus form a broad, mostly flat region while in sheep, their curvature is more complex and the posterior slope is greater. This makes it easier to define RP1 in humans as the posterior beginning of the tibial plateau. The intercondylar eminence, consisting of the medial and lateral tibial spines, is more symmetrical in humans^6,8^, altering the shape and distance of the structures corresponding to RP2-3. The area intercondylaris anterior has a marked protrusion in sheep, unlike in humans. In sheep, the proximal end of the tibial tuberosity is at the level of the tibial plateaus, making them appear more triangular compared to the more oval human ones^7^. Due to the origin of the tendon of the extensor digitorum longus muscle^7,8^, a very pronounced groove is present in the anterolateral part of the ovine plateau^6^ precisely marking RP5 in sheep, missing in humans. The insertion sites of the meniscal roots and cruciate ligaments also differ between the two species^6^, although not relevant when identifying these reference points.

Objectively distinguishing between a normal and OA osteochondral unit is essential for translational and clinical settings. Statistical shape modelling (SSM) is increasingly used in computer-aided surgery, describing the complex geometry and natural variability of three-dimensional anatomical structures like bones and joints^43–48^, with the possibility of incorporating other parameters such as density into the model^44,49^. SSMs may help to reconstruct patient-specific anatomy from partial or unclear anatomical information and can be used to assess variations in shape to detect gross pathological alterations during knee OA^50^. However, it is unclear whether minute changes (without radiographic OA^51^) that are detectable only by meticulous macroscopical observation (i.e. early OA lesions^52^) may be noticed with such an automated method.

3-D registration of the image sets also provides a powerful tool to compare identical regions of different samples^18^, or the same scene taken at different time points^19^. Landmark-based registration approaches are attractive because their interpretation is easy and intuitive, and solutions can be computed fast and efficiently^19^. In the registration of bony structures usually rigid (translation and rotation), similarity (translation, rotation and uniform scaling), and affine (translation, rotation, scaling and shear) transformations are used^18^. The five reference points defined here together or in combination with other landmarks can be used as landmarks for this technique, providing a simple, standardized way to register ovine tibial plateau samples either with rigid or affine registration algorithms, thus enabling a direct comparison between OA and matching control samples.

This study has certain limitations. The allocation of RL6-11 is relatively cumbersome due to the requirement of using ROIs on the micro-CT sections. As aimed to specifically define bony landmarks, the peripheral region does not entirely correspond to the area covered by the menisci^25^. Likewise, a separation of a region corresponding to the beginning of the diffuse upward concavity of the tibial spines within the central region^25^ was not attempted here because of the lack of a specific bony landmark. Moreover, the landmark-based registration based on the new reference points was chosen to be performed on lower resolution micro-CT datasets, a challenge primarily due to the huge amount of data generated with micro-CT compared to clinical CT scanners, with which proximal tibias are scanned with about 130 µm voxel size^12^, resulting in a much lesser number of images. Strengths are the definition of subregions of each tibial plateau based on bony landmarks and topographical allocations of normal values of the osteochondral unit and pathological phenomena such as OA cartilage lesions and osteophytes, together with the 3-D image registration. This provides a strong basis for translational investigations to define the exact location of histological and biochemical sample harvesting, biomechanical tests and any anatomical points of interest in a reproducible way.

In summary, this study defines 5 reference points on the ovine tibial plateau, which are convenient to recognize both macroscopically and in micro-CT 2-D coronal sections. These reference points may serve as standardized landmarks for landmark-based image registration. Characterization of their identification revealed a high intra- and interobserver reliability. The ability to divide a tibial plateau into 6 subregions allows for a precise definition of the location of identical sample harvesting for different analyses. Finally, topographical thickness measurements of the osteochondral unit and mapping the presence of pathological phenomena such as OA cartilage lesions and osteophytes in a fashion relevant for translational investigations are possible using this approach.

## Materials and Methods

### Study design

**Figure 8:**
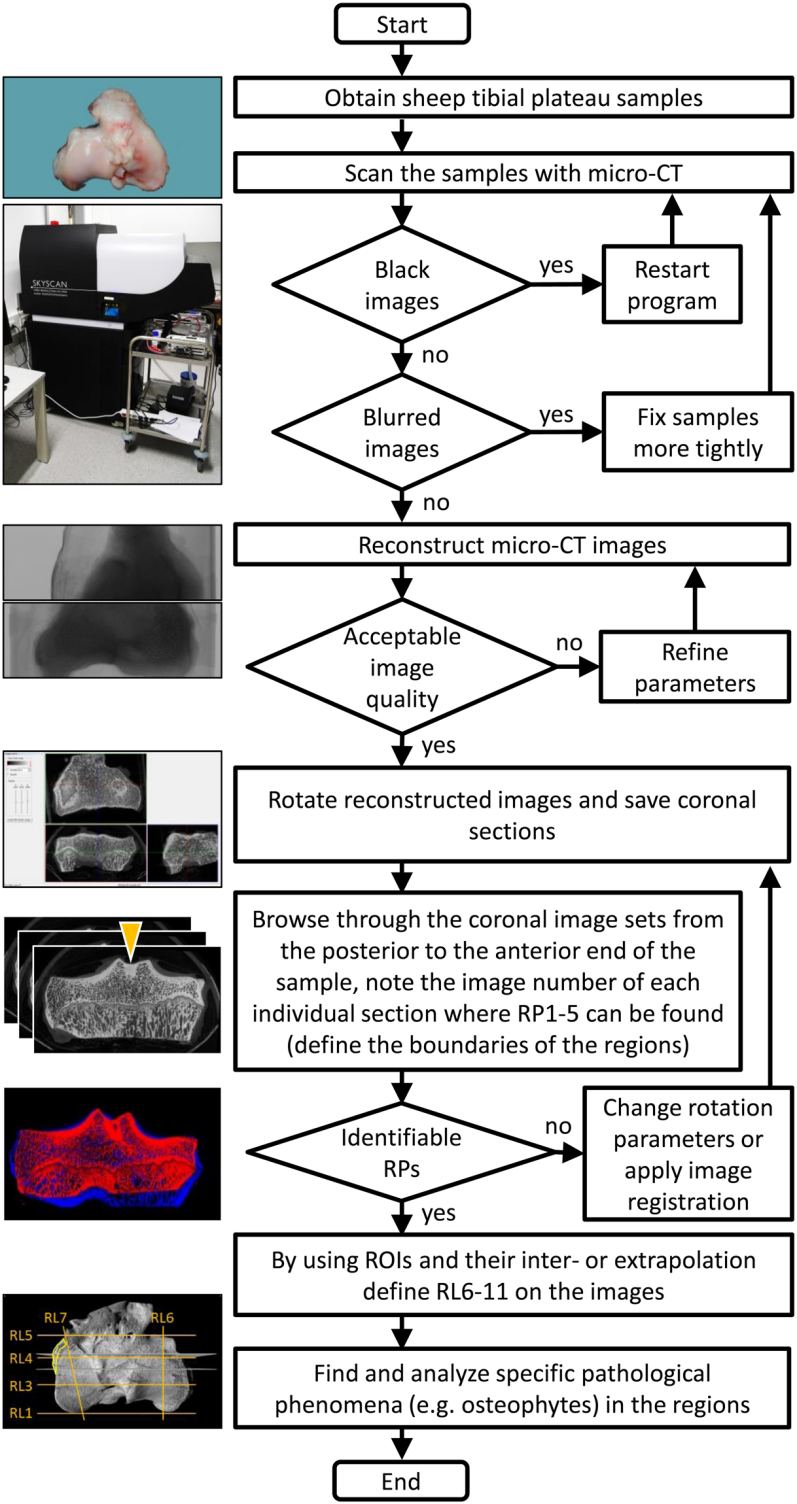
Flowchart diagram of the workflow to identify specific pathological phenomena. The individual steps required for obtaining micro-CT image sets, and their analysis for determining the location of specific pathological phenomena including osteophytes using the newly defined regions are illustrated. A particularly important step is the uniform rotation of the reconstructed images.

Eighteen sheep tibial plateaus (9 left; 9 right) from a previous study were scanned by micro-CT. Five reference points were determined on each sample (Fig. 8). To test the sensitivity of the relative distance of the reference points to rotation, the scanned and reconstructed images of 8 left tibial plateaus were rotated in the sagittal and axial planes by + and −10 degrees, and the reference points were determined on coronal sections of the rotated images. Both intra- and interobserver reliabilities were determined for all of the defined five reference points.

### Animal experiments

Nine healthy Merino ewes (mean body weight, 70 ± 20 kg; age ~1.5 years old) underwent open transection of the anterior root of the medial meniscus on their right knees^53^ for an unrelated study. Their left knees served as unoperated control. All animal experiments were conducted in agreement with the national legislation on protection of animals and the National Institutes of Health (NIH) Guidelines for the Care and Use of Laboratory Animals (NIH Publication 85–23, Rev 1985) and were approved by the Saarland University Animal Committee according to German guidelines. Animals were sacrificed at 6 months postoperatively. Tibial plateaus were harvested and used for micro-CT evaluations. Artefacts visible on some of the images are related to experiments not described here, and they are not affecting the reliability of the present study.

### Micro-CT imaging

Tibial plateau specimens (n = 18) were scanned in a microfocused x-ray computed tomography (micro-CT) scanner (Skyscan 1176, Bruker microCT, Kontich, Belgium) as described previously^13,54^. The device possesses a moveable 11-MP camera and an x-ray tube, allowing for a spatial resolution up to 9 μm. For the acquisition of 12-bit x-ray shadow transmission images, the tube voltage was set at 90 kV, and the current was 278 µA. All specimens were scanned at a spatial resolution of 18 µm pixel size. Projections were obtained with a combined 0.5-mm aluminium/copper filter interposed, at 0.4° intervals with 270-millisecond exposure time, by averaging 3 frames at each position. Images were reconstructed by a modified Feldkamp cone-beam algorithm^55^ (NRecon software v. 1.7.0.4., Bruker microCT). Smoothing, misalignment correction, ring artefact reduction, and beam hardening correction was applied (2, 1, 10, 30%, respectively, no units) during the reconstruction. Grey values were set between −0.000023 and +0.020154 for optimal representation of bony parameters.

The uniform rotation of the reconstructed datasets (DataViewer software v. 1.5.2.4., Bruker microCT) prior to saving the coronal section images was crucial to obtain comparable image sets. The groove of the extensor digitorum longus muscle (see Fig. 1) which can always be found in the anterior lateral region is a landmark of great importance to distinguish the medial, lateral, anterior and posterior parts of the tibial plateaus on the 2-D image sets. In the DataViewer software, the following criteria should be fulfilled: 1) in the window showing the axial plane (top view), the posterior ends of the tibial plateaus have to end at the same imaginary line, parallel with the frontal plane, 2) in the window showing the sagittal plane (lateral view), in a position between the tibial spines, the two peaks of the growth plate (if both of them are visible) and the plane of the tibial plateau have to be as horizontal as possible, and 3) in the window showing the coronal plane (frontal view), the medial and the lateral tibial plateaus in the intermediate part have to be as horizontal as possible.

For samples whose manual rotation is problematic to provide identical alignment to the other samples, image registration in DataViewer can be applied. After an inadequate rotation, the coronal section image set was resized and saved in the CTan software (version 1.16.4.1., Bruker microCT), to obtain images with significantly decreased final resolution (approximately 500 × 300 pixels) suitable for faster image processing. The same process was repeated on an image set which was already rotated to the desired position, to provide a “reference” image set for the registration. Both image sets were loaded in the 3D registration option of the DataViewer software, and true 3D registration was performed. DataViewer supports rigid transforms containing x/y/z translations, 3D rotation and re-sampling, and uses sum of square difference as criteria. From the log file of the “target” image set (the sample with the originally inadequate rotation), the parameters of the rotations were applied for the full-resolution image set of the same sample to achieve identical rotation to that of the reference sample.

Coronal sections were used for further evaluations (Fig. 8). Image sets of the individual samples consisted of 1843 ± 76 sections with a mean resolution of 3515 ± 80 × 2255 ± 171 pixels (n = 18). A screening of all coronal micro-CT images allowed a precise identification of the defined reference points. This analysis was performed from the posterior to the anterior direction in order to identify the reference points, starting with RP1.

For 3-D reconstruction of the micro-CT image sets the CTVox v. 3.2.0. (Bruker microCT) program was used, employing shadows and surface lighting to enhance the visibility of the surface structures of the samples.

### Intraobserver reliability

For intraobserver comparison, the data were taken from the same database and analysed twice on separate occasions with a 3-week-long delay between Trial 1 and Trial 2, by the same observer (TO). During the analysis, the researcher browsed through the micro-CT 2-D coronal sections from the posterior end to the anterior end of the sample, and noted the numbers of those individual images on which the reference points were located in their most characteristic views. The 5 reference points were determined on the same 18 samples (9 left and 9 right tibial plateaus).

### Interobserver reliability

For testing the interobserver reliability of the reference points, 3 observers (TO, LG, JR) with different experience in micro-CT analysis, blinded to each other, were asked to identify the 5 reference points on 2-D coronal image sets of 8 left tibial plateaus. The image number of each reference points was recorded in a standardized Excel table.

### Sensitivity to rotation

Sensitivity of the relative position of the reference points to rotation of the samples was also tested. Reconstructed images of 8 left tibial plateaus were rotated in the DataViewer program in the sagittal and axial planes by + and −10 degrees compared to their original position (0°). Due to the marked changes in the total image numbers of the image sets due to rotations, and to a slight inter-specimen variability of the total length of the tibial plateaus, the relative positions of the reference points were used for analysis and presentation. RP1, by definition, as the posterior tip of the medial tibial plateau, was considered as 0%, while RP5 was defined as 100% in each sample. The relative positions of RP2-4, expressed in percentages, were determined for each rotation.

### Assessing the usability of the subregions

To assess the usability of the newly defined subregions, samples were harvested from pre-defined parts of left tibial plateaus, and were stained with Safranin O / fast green according to standard protocols^56^. Right sheep knees (n = 9) always underwent an open transection of the anterior root of the medial meniscus^53^. All tibial plateaus were examined for the presence of osteophytes and the extent of OA lesions. Based on India ink staining of the samples^25^, the area affected by OA lesions was classified into 4 categories: 0–25%, 25–50%, 50–75%, 75–100% of the affected area of the individual subregions. For topographic mapping of the subchondral bone plate thickness and the cartilage thickness, RL1-11 were identified on an example 2-D micro-CT image set of a randomly chosen control sheep knee. The above parameters were determined on 3-3 slices equidistant from each other within each subregion (altogether 98 measurement points within a sample), and plotted on the 3-D reconstructed image of the sample.

### Assessing the usability of the reference points for 3-D image registration

Resolution of coronal section micro-CT image sets of two randomly chosen left tibial plateau samples were decreased to 453 × 223 and 594 × 364 pixels in the CTan software to make them suitable for faster image processing. For landmark-based rigid registration the 3D Viewer^57^ plugin of Fiji^58^ (ImageJ version 1.51n) was used. For landmark-based affine registration the 3D Slicer program (version 4.6.2)^59^ with the SlicerRT plugin (version 0.18.0)^60^ was used. RP1-5 were selected on the 2-D micro-CT image sets of both samples in the programs, and registration was performed. Accuracy of the affine registration with additional 2 landmarks selected on the medial and lateral peaks of the tibial plateau on the 2-D section corresponding to RL3 was also tested. Adequacy of the registration was verified with visual inspection of the resulting 2-D and 3-D image sets.

### Statistical analysis

To test the intra- and interobserver reliability of finding the same reference points on the same image numbers, intraclass correlation coefficients (ICC) were determined^61^ with the MedCalc 17.4 program (MedCalc Software, Ostend, Belgium) using the following settings: two-way model, the same raters for all subjects, type: absolute agreement. For this analysis, the raw image numbers of micro-CT slices showing the reference points were used, since they had the best comparability. However, for better presentation and easier interpretation of the data, the image numbers were converted to mm-s, and the distances corresponding to the blank images occasionally present at the beginning of the datasets were subtracted. For another determination of reliability, the total lengths of the tibial plateaus (between RP1 and RP5) were calculated. The intra- and interobserver differences (i.e. the errors) were calculated in percentages of the total tibial plateau length. The results were deemed acceptable if these errors were less than 5%^62^.

Statistical significance in the relative positions of the reference points between the different rotations and the original samples was tested by one way repeated measures analysis of variance (RM ANOVA) or non-parametric RM ANOVA with SigmaPlot, version 12.0 (Systat Software, Inc., San Jose, CA, USA). In all tests, *P* < 0.05 was considered statistically significant.

All data are expressed as mean ± standard error of the mean (SEM).
